# Psychiatric and psychosocial problems in adults with normal-intelligence autism spectrum disorders

**DOI:** 10.1186/1471-244X-9-35

**Published:** 2009-06-10

**Authors:** Björn Hofvander, Richard Delorme, Pauline Chaste, Agneta Nydén, Elisabet Wentz, Ola Ståhlberg, Evelyn Herbrecht, Astrid Stopin, Henrik Anckarsäter, Christopher Gillberg, Maria Råstam, Marion Leboyer

**Affiliations:** 1 Forensic Psychiatry, Department of Clinical Sciences, Malmö, Lund University, Lund, Sweden; 2 INSERM, U 995, dept of Genetics, Institut Mondor de Recherche Biomédicale, Psychiatry Genetics, Creteil, France; 3 Child and Adolescent Psychiatry, Institute of Neuroscience and Physiology, University of Gothenburg, Gothenburg, Sweden; 4 Vårdal Institute, Swedish Institute for Health Sciences, Lund, Sweden; 5 Forensic Psychiatry, Institute of Neuroscience and Physiology, University of Gothenburg, Gothenburg, Sweden; 6 Assistance Publique-Hôpitaux de Paris, Henri Mondor-Albert Chenevier Hospitals, Department of Psychiatry, Creteil, France; 7 Fondation FondaMental, Creteil, France; 8 Department of Clinical Sciences, Lund, Child and Adolescent Psychiatry, Lund University, Lund, Sweden; 9 University Paris 12, Faculty of Medicine, IFR10, Creteil, France

## Abstract

**Background:**

Individuals with autism spectrum disorders (ASDs) often display symptoms from other diagnostic categories. Studies of clinical and psychosocial outcome in adult patients with ASDs without concomitant intellectual disability are few. The objective of this paper is to describe the clinical psychiatric presentation and important outcome measures of a large group of normal-intelligence adult patients with ASDs.

**Methods:**

Autistic symptomatology according to the DSM-IV-criteria and the Gillberg & Gillberg research criteria, patterns of comorbid psychopathology and psychosocial outcome were assessed in 122 consecutively referred adults with normal intelligence ASDs. The subjects consisted of 5 patients with autistic disorder (AD), 67 with Asperger's disorder (AS) and 50 with pervasive developmental disorder not otherwise specified (PDD NOS). This study group consists of subjects pooled from two studies with highly similar protocols, all seen on an outpatient basis by one of three clinicians.

**Results:**

Core autistic symptoms were highly prevalent in all ASD subgroups. Though AD subjects had the most pervasive problems, restrictions in non-verbal communication were common across all three subgroups and, contrary to current DSM criteria, so were verbal communication deficits. Lifetime psychiatric axis I comorbidity was very common, most notably mood and anxiety disorders, but also ADHD and psychotic disorders. The frequency of these diagnoses did not differ between the ASD subgroups or between males and females. Antisocial personality disorder and substance abuse were more common in the PDD NOS group. Of all subjects, few led an independent life and very few had ever had a long-term relationship. Female subjects more often reported having been bullied at school than male subjects.

**Conclusion:**

ASDs are clinical syndromes characterized by impaired social interaction and non-verbal communication in adulthood as well as in childhood. They also carry a high risk for co-existing mental health problems from a broad spectrum of disorders and for unfavourable psychosocial life circumstances. For the next revision of DSM, our findings especially stress the importance of careful examination of the exclusion criterion for adult patients with ASDs.

## Background

Autism spectrum disorders (ASDs) (or pervasive developmental disorders (PDDs), in the DSM-IV) are impairing developmental disorders characterized by aberrations in the domains of social interaction, communication and stereotyped or repetitive behavior patterns, estimated to affect about 1% of the general population [1]. The DSM-IV includes the following ASDs: autistic disorder (AD) (pervasive problems/deficits in all three domains), Asperger's disorder (AS) (pervasive deficits in social interaction and in behaviours in the presence of a normal verbal development) and pervasive developmental disorder not otherwise specified (PDD NOS). Research criteria for AS by Gillberg & Gillberg (G & G) [2] include the same triad of restrictions but also verbal peculiarities and abnormal motor development. "High-functioning autism" (HFA) is a disputed term sometimes used to describe individuals with AD without concomitant mental retardation [3].

Community-based studies show highly skewed male>female ratios for ASDs [4]. Possible sex differences in the clinical phenotypes have been insufficiently studied [5], and instruments and criteria have been developed and validated mostly on male subjects, which also might have affected the estimated sex ratio. Further, ASDs have mainly been diagnosed among children and adolescents, but increasing attention is directed to their prevalence and clinical presentation among adults. A few long-term prospective follow-up studies have so far shown high diagnostic stability [6,7].

Data on psychosocial life circumstances and psychiatric comorbidity in normal-intelligence adult patients with ASDs are scarce but suggest reduced social functioning, and a substantially better outcome in AS than in autism, probably attributable to better intellectual abilities [7]. Estimated rates of co-existing psychiatric disorders in subjects with normal intelligence ASDs have varied substantially, from 9% to 89% [8]. Attention deficits and hyperactivity have been shown to be common in children with ASDs [4], but studies of the co-occurrence of ADHD and ASDs in adults are few [9,10]. A high rate of chronic tic disorders has been reported in ASD patients [11]. Mood disorders, together with anxiety disorders, have been described as important complications of ASDs in a range of studies [8,12,13].

Autism was until the 1970's conceptualized as the earliest manifestation of schizophrenia [14]. Kolvin [15], among others, provided evidence of a bimodal distribution of onset in "childhood psychosis", which was thought to separate the two conditions. It was even suggested that at least the childhood-onset subtype of schizophrenia was less common in autism than in the general population [16]. Today, autism and schizophrenia are referred to as early and late onset neurodevelopmental disorders [17]. Psychotic symptoms in ASD patients have often come to be regarded as misattributions of autistic phenomena [18]. However, "schizophrenic-type illnesses" represent around one-tenth of all psychiatric comorbid diagnoses in a review by Howlin [8]. Additionally, a number of clinical case reports have described psychotic symptomatology, including auditory hallucinations, paranoid ideas, or delusional thoughts in subjects with ASDs. It seems probable that ASD is one possible vulnerability factor for the development of psychotic symptoms and schizophrenia [19]. A more definitive picture of the life-time prevalences of ASD, psychotic disorders and their overlap will require population-based prospective studies.

We have compiled detailed data on a large group of consecutively referred adults diagnosed with normal-intelligence ASDs to: 1. detail the criteria for the various problem types in the DSM-IV ASD subgroups and between male and female subjects. 2. estimate frequencies of DSM-IV axis I and II diagnoses and describe their diagnostic overlap in adults with normal-intelligence ASDs and 3. explore the psychosocial situation for these subjects.

## Methods

Participants in this study were consecutively referred adults with possible childhood-onset neuropsychiatric disabilities at the Henri Mondor-Albert Chenevier hospital in Paris ("the Paris study group") and at the Child Neuropsychiatric Clinic in Gothenburg ("the Gothenburg study group") who subsequently met DSM-IV criteria for an ASD with normal intelligence. Both clinics are expert diagnostic centers focused on neuropsychiatric assessments of childhood-onset disorders in adults. The Paris site was specifically recruiting patients with AS and other ASDs. For this study, eight patients were also included according to the Paris protocol at the Psychiatric Outpatient Clinic in Malmö.

The total study group of 122 adults (39 from Paris and 83 from Gothenburg) included 82 (67%) men and 40 (33%) women (median age 29 years (yrs), ranging from 16 to 60 yrs). The Gothenburg subjects were significantly older than the Paris subjects (Mann-Whitney U = 1072, p = 0.003) with a median age of 30 yrs (range 19–60 yrs) as compared to 25 yrs (range 16–47 yrs) in Paris. There were no significant differences in sex ratios (χ^2 ^= 1.33, df = 1, p = 0.30) or full scale IQ (Mann-Whitney U = 1170, p = 0.46) between the two study groups. In Gothenburg 2% of cases were diagnosed with AD, 46% with AS and 52% with PDD NOS. Among the Paris subjects, AD was diagnosed in 8%, AS in 74%, and PDD NOS in 18%. The difference in frequency of AS and PDD NOS diagnoses between Gothenburg and Paris were significant (χ^2 ^= 8.75, df = 1, p = 0.004 and χ^2 ^= 12.58, df = 1, p < 0.001). Men and women in the total study group did not differ significantly in age (Mann-Whitney U = 1392, p = 0.18) with the median age being 28 yrs for men and 30 yrs for women. Sex differences within the diagnostic ASD subgroups did not reach significance, as was the case in another study of an adult psychiatric population [10].

The Gothenburg study group includes patients diagnosed with ASD from a previously described study group of adults with childhood-onset neuropsychiatric disorders [9,20]. All subjects were seen on an outpatient basis by clinicians involved in autism research (all included patients had their final diagnoses confirmed by either HA, MR or ML). The individual diagnoses were based on all available information, including current clinical status. Childhood developmental problems were assessed retrospectively, from direct parental report where possible. More than half of the subjects had earlier been diagnosed with anxiety, mood disorders or psychosis and were now secondary or tertiary referrals from specialists in adult psychiatry for additional diagnostic work-up. Childhood medical records, including previous psychiatric or psychological assessments, were provided by the patients or obtained from child medical centers. All subjects were included according to the research protocol for the Gothenburg Neuro-Psychiatry Genetics Project (NPG) or the Paris Autism Research International Sibpair study (P.A.R.I.S.).

The Asperger Syndrome Diagnostic Interview (ASDI) [21] was used in 106 subjects (87%). ASD diagnoses were assigned according to specific assessments of all DSM-IV autistic disorder criteria and the Gillberg & Gillberg (G&G) criteria for AS [2]. In the Gothenburg group, 63 patients (76%) had axis-I disorders assessed by the Structured Clinical Interview for DSM IV – Axis I Disorders (SCID-I) [22]; all other subjects had a structured, DSM-IV-based, clinical interview supplemented with a life-time DSM-IV symptom checklist containing individual criteria or symptom definitions for all relevant axis-I disorders. Axis-II disorders were assessed in 117 patients (96%), in 95 subjects (81%) by the Structured Clinical Interview for DSM IV – Axis II Personality Disorders (SCID-II) [23] and in the others by a structured DSM-IV-based clinical interview. For all disorders, DSM criteria that limited the possibility of assigning other comorbid psychiatric diagnoses were disregarded to allow a comprehensive recording of the pattern of comorbidity.

Somatic status was assessed in all patients. Cases with known medical causes of autism, including genetic syndromes, or injuries of relevance for the mental disorders assessed, were excluded by history, physical examination, and in dubious cases by karyotype, Fragile × PCR and southern blot, and FISH analyses (15q11-q13, 22q11 and 22q13 deletion syndromes). No patient was in need of language interpretation for communication. Three-generation pedigrees were drawn. In the Gothenburg study group, whenever possible, a semi-structured collateral interview (n = 63, 76%) based on the ASDI, the ADHD-RS [24], the "Five to Fifteen" questionnaire [25], and the Wender Utah Rating Scale [26] was performed with a relative who had known the index subject as a child. In the Paris study group the Autism-Tics, ADHD and Other Comorbidities Inventory (A-TAC) [27], was used for collateral interviews (n = 39, 100%). Global intellectual ability was assessed in most cases (n = 114, 93%) using the Wechsler Adult Intelligence Scale-Revised (WAIS-R) (n = 83) or the Wechsler Adult Intelligence Scale-III (WAIS-III) (n = 31) [28,29]. The remaining eight subjects either had normal results from previous tests or well-documented normal development according to school and educational performance but declined participation in new psychometric assessments.

The study was approved by the medical ethical review boards at each site (Gothenburg, Paris and Malmö). All patients included gave written informed consent.

Statistical analyses were performed using the SPSS 15.0 [30]. Since AD was diagnosed only in five subjects, these subjects were described separately and not included in group comparisons between diagnostic groups. Mann-Whitney U test was used for group comparisons of differences in continuous variables, as the data was not normally distributed. Fisher's exact χ^2^-test was used to compare differences in frequencies of fulfilled ASD, DSM-IV and G & G criteria and coexisting psychopathologies. All p-values are two-tailed, and significance was considered at the 5% level.

## Results

### Autism Spectrum Disorders (Pervasive Developmental Disorders)

The distribution of DSM-IV and G & G criteria across the diagnostic categories and sexes is presented in Table 1. Virtually all subjects (n = 119, 98%) displayed symptoms required for the first DSM-IV and G & G criterion (i.e. social interaction problems, in the DSM-IV also including non-verbal communication deficits). Nonverbal communication problems according to the fifth G & G criterion were very common, described in 89% (n = 108) of all subjects. The AS and the PDD NOS subjects did not differ significantly in the DSM-IV and G & G areas of social interaction and the DSM-IV area of communication.

**Table 1:** Distribution of DSM-IV and Gillberg and Gillberg (G&G) criteria across the diagnostic categories

Type of DSM-IV PDD	Autistic disorder (N=5)		Asperger's disorder (N=67)		PDD NOS (N=50)		AS – PDD NOS^a^		Total (N=122)		Male (N=82)		Female (N=40)		Male – Female^a^	
	
	N	%	N	%	N	%	χ^2 ^ (df = 1)	p	N	%	N	%	N	%	χ^2 ^ (df = 1)	p
DSM IV criterion A1 "Qualitative impairment in social interaction"	5	100	67	100	47	94	4.13	0.08	119	98	80	98	39	98	0.00	1.00
																
DSM-IV Criterion A2: "Qualitative impairment in communication"	5	100	34	51	18	36	2.52	0.13	57	47	41	50	16	40	1.08	0.34
																
DSM-IV Criterion A3: "Restricted, repetitive and stereotyped behaviour, interests, and activities"	5	100	65	97	29	58	27.60	<0.001	99	81	70	85	29	73	2.91	0.14
																
G&G Criterion 1; "Social interaction problems"	5	100	67	100	47	96	4.13	0.08	119	98	80	98	39	98	0.00	1.00
																
G&G Criterion 2: "Narrow interests"	5	100	64	96	33	67	17.61	<0.001	102	84	69	84	33	83	0.05	0.80
																
G&G Criterion 3; "Repetitive routines"	5	100	61	91	25	51	24.77	<0.001	91	75	62	76	29	73	0.14	0.83
																
G&G Criterion 4: "Speech and language"	5	100	56	84	22	45	20.19	<0.001	83	69	57	70	26	65	0.25	0.68
																
G&G Criterion 5: "Non-verbal communication problems"	5	100	66	99	37	76	16.33	<0.001	108	89	74	90	34	85	0.73	0.38
																
G&G Criterion 6: "Motor clumsiness"	5	100	57	85	31	63	8.18	0.005	94	78	60	73	33	83	1.29	0.37

^a^Fisher's exact *χ*^2 ^test

### Other Axis I Psychiatric Disorders "Usually First Diagnosed in Infancy, Childhood, or Adolescence"

A large proportion of all subjects was diagnosed with ADHD (n = 52, 43%, Table 2). Subjects with PDD NOS had significantly more symptoms of inattention (Mann-Whitney U = 1157, p = 0.01) and hyperactivity/impulsivity (Mann-Whitney U = 1136, p = 0.007) compared to subjects with AS. However, the prevalence of the categorical diagnosis of ADHD did not differ significantly between the groups.

**Table 2:** Frequency of ADHD subtypes and symptoms of inattention and hyperactivity/impulsivity in each of the ASD subtypes

	Autistic disorder (N=5)		Asperger's disorder (N=67)		PDD NOS (N=50)		AS – PDD NOS^a^		Total (N=122)		Male (N=82)		Female (N=40)		Male – Female^a^	
	
	N	%	N	%	N	%	χ^2 ^ (df = 1)	p	N	%	N	%	N	%	χ^2 ^ (df = 1)	p
Inattentive subtype	2	40	8	12	11	22	2.13	0.21	21	17	14	17	7	18	0.00	1.00
Hyperactive/impulsive subtype	0	0	5	8	3	6	0.10	1.00	8	7	5	6	3	8	0.09	0.72
Combined subtype	0	0	11	16	12	24	1.04	0.35	23	19	16	20	7	18	0.07	1.00
Any ADHD	2	40	24	36	26	52	3.06	0.09	52	43	35	43	17	43	0.00	1.00

							AS – PDD NOS^b^								Male – Female^b^	
	
							Z	p							Z	p

Median and range of inattentive criteria met	4 (0–7)		3 (0–8)		6 (0–9)		-2.52	0.01	4 (0–9)		4 (0–9)		3 (0–9)		-0.49	0.63
Median and range of hyperactive/impulsive criteria met	2 (0–7)		1 (0–9)		3 (0–9)		-2.69	0.007	2 (0–9)		2 (0–9)		2 (0–9)		-0.89	0.37

^a^Fisher's exact *χ*^2 ^test; ^b^Mann-Whitney U test

The frequency of reading disorder in combination with disorder of written expression (i.e. dyslexia) was 14% (n = 16). In the Gothenburg group the criteria for this diagnosis was an unambiguous history of deficient reading and writing; the Paris subjects had a formal diagnosis of dyslexia.

### Adult Axis I Disorders

The frequencies of the remaining DSM-IV axis-I diagnoses are presented in Table 3. Among the small number of subjects with AD, 80% (n = 4) met criteria for at least one other major axis-I disorder as specified below. In the AS and PDD NOS subgroups all subjects had at least one comorbid axis-I disorder. The most common life-time comorbid condition was mood disorder (n = 65, 53%). One-third of subjects (n = 42, 34%) had been treated with an antidepressant at least once in their lives. Criteria for a bipolar disorder (BP) were met by 10 subjects (8%), five of whom had bipolar I subtype and two bipolar II, while three were coded as unknown subtypes. No subject with AD met criteria for BP. Only three patients (2%) had ever been treated with a mood stabilizer.

**Table 3:** Lifetime rate of axis-I disorders in adults with autism spectrum disorders (N = 122, if not otherwise specified)

	Criteria met DSM-IV															
	
	Autistic disorder (N=5)		Asperger's disorder (N=67)		PDD NOS (N=50)		Total (N=122)		AS – PDD NOS^a^		Male (N=82)		Female (N=40)		Male – Female^a^	
	
	N	%	N	%	N	%	N	%	χ^2^ (df = 1)	p	N	%	N	%	χ^2 ^ (df = 1)	p
Attention-Deficit/Hyperactivity Disorder	2	40	24	36	26	52	52	43	3.06	0.09	35	43	17	43	0.00	1.00
Chronic tic disorders	0	0	14	21	11	22	25	20	0.02	1.00	20	24	5	13	2.33	0.16
Mood disorder	3	60	35	52	27	54	65	53	0.04	1.00	39	48	26	65	3.29	0.08
Psychotic disorders	0	0	10	15	5	10	15	12	0.62	0.58	13	16	2	5	2.94	0.14
Substance related disorders	1	20	4	6	14	28	19	16	10.67	0.002	14	17	5	13	0.43	0.60
Anxiety disorder N = 119	0	0	34	51	25	50	59	50	0.01	1.00	37	45	22	55	1.05	0.34
Obsessive Compulsive Disorder	0	0	14	21	15	30	29	24	1.27	0.29	16	20	13	33	2.50	0.12
Impulse control disorder	0	0	4	6	7	14	11	9	2.17	0.20	6	7	5	13	0.88	0.50
Somatoform disorder N = 119	0	0	2	3	4	8	6	5	1.48	0.40	4	5	2	5	0.00	1.00
Eating disorder N = 119	0	0	2	3	4	8	6	5	1.48	0.40	2	2	4	10	3.29	0.09

^a^Fisher's exact *χ*^2 ^test

A considerable number of patients (n = 15, 12%) met criteria for a psychotic disorder (most often not otherwise specified). Four patients met criteria for a schizophreniform disorder, three for brief psychotic disorder, and one for a delusional disorder. No subject met criteria for schizoaffective disorder. In the entire study group, 18 subjects (15%) had been treated with neuroleptics at least once in their lives.

Sixteen per cent of the subjects (n = 19) met criteria for a substance use disorder (SUD). The PDD NOS group had significantly more SUD-related diagnoses than the AS group (p = 0.002). The majority of diagnoses were related to alcohol (n = 15, 12%), four subjects met criteria for cannabis use disorder, three for amphetamine use disorder, two had a history of taking non-prescribed opiates or analgetics, and one had used anabolic steroids. Another subject, a 27-year-old man with AD, had a history of inhaling solvents.

The second most frequent category of DSM-IV disorders was anxiety disorders. Generalized anxiety disorder was common (n = 18, 15%) as was social phobia (n = 16, 13%). Thirteen subjects (11%) met criteria for panic disorder and/or agoraphobia and seven (6%) met criteria for a specific phobia. Two patients suffered from post traumatic stress disorder (PTSD), and one had an anxiety disorder NOS.

Among patients affected with impulse control disorders, intermittent explosive disorder was the most common diagnosis (n = 7, 6%), followed by kleptomania, pyromania, pathological gambling, trichotillomania, and impulse control disorder NOS, all affecting one patient each.

### Personality disorders

Rates for personality disorders (PD) according to DSM-IV are presented in Table 4. Obsessive-compulsive PD (OCPD) was significantly more common in the AS group (χ^2 ^= 4.26, df = 1, p = 0.04) and antisocial PD in the PDD NOS group (χ^2 ^= 5.14, df = 1, p = 0.04). Overall frequency of PDs did not differ between men and women, with the exception of schizoid PD, which was significantly more common among the female subjects (χ^2 ^= 6.72, df = 1, p = 0.02).

**Table 4:** Lifetime rate of axis-II disorders in adults with autism spectrum disorders (N = 117)

	Criteria met DSM-IV															
	
	Autistic disorder (N=5)		Asperger's disorder (N=62)		PDD NOS (N=50)		Total (N=117)		AS – PDD NOS^a^		Male (N=77)		Female (N=40)		Male – Female^a^	
	
	N	%	N	%	N	%	N	%	χ^2 ^(df = 1)	p	N	%	N	%	χ^2 ^(df = 1)	p
Personality disorders																
≥ 1 PD	1	20	42	68	30	60	73	62	0.72	0.43	46	60	27	68	0.68	0.43
≥ 2 PD	0	0	25	40	16	32	41	35	0.83	0.43	28	36	13	33	0.18	0.84
≥ 3 PD	0	0	11	18	9	18	20	17	0.00	1.00	13	17	7	18	0.01	1.00
Paranoid	0	0	12	19	10	20	22	19	0.01	1.00	15	20	7	18	0.07	1.00
Schizotypal	0	0	10	16	5	10	15	13	0.90	0.41	12	16	3	8	1.54	0.26
Schizoid	0	0	13	21	12	24	25	21	0.15	0.82	11	14	14	35	6.72	0.02
Histrionic	-	-	-	-	-	-	0	0	-	-	-	-	-	-	-	-
Narcissistic	0	0	1	2	2	4	3	3	0.61	0.59	2	3	1	3	0.00	1.00
Borderline	0	0	4	7	6	12	10	9	1.05	0.34	4	5	6	15	3.24	0.09
Antisocial	0	0	0	0	4	8	4	3	5.14	0.04	3	4	1	3	0.16	1.00
Avoidant	0	0	18	29	11	22	29	25	0.62	0.52	21	28	8	20	0.81	0.50
Dependent	0	0	2	3	4	8	6	5	1.24	0.41	3	4	3	8	0.70	0.41
Obsessive	1	20	25	40	11	22	37	32	4.26	0.04	23	30	14	35	0.32	0.68
PD NOS	-	-	-	-	-	-	0	0	-	-	-	-	-	-	-	-

^a^Fisher's exact *χ*^2 ^test

### Psychosocial Characteristics

A majority of the subjects (n = 68, 56%) reported that they had been bullied at school. Such victimization was most common among the women (χ^2 ^= 6.09, df = 1, p = 0.02). The educational level was relatively high in the entire study population. Two thirds (n = 77, 65%) had graduated from upper secondary school, and a quarter (n = 29, 24%) had completed college or university studies. In terms of daily occupation, 43% (n = 50) were employed or were students at the time of the assessment, with no significant differences between males and females. The others had either no organized daily activities, were on sick leave, held a medical pension, or were unemployed. Half of the subjects aged 23 yrs or more had independent living arrangements, as did some of the younger subjects. Nineteen (16%) had lived in a long-term relationship. Men and women did not differ in terms of marriage or cohabitation.

## Discussion

Large outcome studies or systematic clinical surveys of adult ASDs are few. To our knowledge, this is one of the first such studies presenting detailed clinical data on a large consecutive group of adults with ASDs and normal intelligence. It includes a wide age span (16–60 yrs), with a relatively large proportion of subjects over 30 yrs of age (42%), and a substantial representation of women.

The purpose of describing the presence of autistic disorder symptoms in all three diagnostic subgroups was to address the important question of the adequacy of the current DSM-IV ASD categories. The interpretation of different patterns of criteria in the three diagnostic groups first has to consider that these criteria were used to assign the diagnoses. Then, as expected, the small group with normal-intelligence AD (equivalent to HFA) had the most pervasive ASD symptomatology, followed by the AS group, while the PDD NOS group exhibited the least number of symptoms. However, one-third of the PDD NOS patients and half of the AS patients met the DSM-IV communication criterion despite the fact that, according to the DSM-IV, only "subtle aspects of social communication" is expected to be impaired in AS, and the criteria for PDD NOS do not even require communication problems. When comparing the distribution of G & G criteria across the subgroups, deficits in the area of "social interaction" were evident in all ASD cases, while the other criteria were all more pronounced in the AS group as compared to the PDD NOS group. A tentative conclusion would be that these findings fit a dimensional model of ASDs and that the high rates for all criteria across the diagnostic categories would speak against their use as differential diagnostic entities.

The proportion of female subjects was high in this consecutively recruited clinical group compared to epidemiological studies [4]. This high representation could suggest that women with ASDs develop more severe social deficits [31] or more concomitant psychopathology. In a group of children and young adults diagnosed with normal-intelligence ASDs, Holtmann and colleagues [5] did not find sex differences in the triad of autism core dysfunctions. Our findings can extend this to an older group of patients.

It is worth noting that referral practices are likely to have enriched our study group with a higher prevalence of comorbid conditions in comparison to the ASD population as a whole. Indeed, many of our patients had previously been in contact with specialists in psychiatry and were then referred to our expert centers. The prevalence of comorbid conditions is also likely to be inflated by our decision to disregard DSM-IV criteria excluding certain diagnoses in the presence of ASD. Nonetheless, this decision was justified by our aim to describe clinical conditions where prevalence would have been zero if strict hierarchical criteria had been followed.

High comorbidity with childhood-onset disorders was expected in our study population. Despite the fact that the current diagnostic classification of ASDs precludes a diagnosis of concomitant ADHD (in DSM-IV) or hyperkinetic disorder (in ICD-10), earlier estimates have reported very high rates of these problems (80–83%) in children with ASDs [4]. In our group, the rate was lower but still substantial. The most common ADHD subtypes were the combined and inattentive forms, which may be due to the different presentation of ADHD in adulthood.

Kanner [32] suggested that the features of the autistic syndrome, for example insistence on sameness, were related to anxiety. Other authors have described patients with ASD as vulnerable to stress because of a restricted repertoire of appropriate coping mechanisms [33]. In agreement with this, anxiety disorders, especially OCD where rates were very similar to a recent study of mostly AS patients [34], were clearly overrepresented as compared to the general population.

Earlier estimates of comorbid depression in autism and AS vary widely, from 4 to 38% [35]. Our high frequency of major depressive disorder might be linked to the higher median age in our study group. This finding and the fact that only a minority of the patients had ever had antidepressant treatment would stress the importance of attention to such symptoms in this patient category. The overlap of symptoms between ASDs and depression (e.g. social withdrawal, impaired non-verbal communication) can make diagnoses difficult, and earlier studies have pointed out the difficulties these patients have in verbalizing their changes in mood and describing depression [36].

Psychotic symptoms in ASDs are controversial. Since our study group was clinically recruited, it cannot be considered to be representative for a general ASD population, but the need for revision of the criteria precluding or diffusing the diagnostic possibilities in this field is obvious.

Substance-related disorders, especially those related to alcohol, were no more common in this group than in the general population, but more prevalent among subjects diagnosed with a PDD NOS than among subjects with AS. This, and the fact that antisocial PD was found only in the PDD NOS group, is in line with other studies describing a subgroup of antisocial individuals having atypical autistic features presenting as PDD NOS [37]

Patients afflicted with ASDs often describe themselves in clinical interviews or in self-rate questionnaires in a way that corresponds to PD characteristics [38]. Our findings, with two-thirds of our subjects meeting criteria for at least one PD, confirm this, as well as a preponderance of OCPD and avoidant PDs [20]. Furthermore, the higher rate of OCPD in AS compared to PDD NOS corresponds to the AS group's higher rate of restrictions in repertoires and interests. However, the AS group did not have a higher rate of OCD as compared to the PDD NOS group. Despite the tendency toward more diagnoses of cluster A and C in the total group, the overall conclusion is that categorical PDs provide a rather unspecific description of the maladaptive patterns of personality function in the ASD group.

A large proportion of the subjects, especially the females, had been bullied during their school years. In spite of high levels of education, more than half of the entire ASD group was unemployed, on sick-leave, or had a medical pension. Some 40% were still living with their parents or in community-based group homes. In line with previous studies, only a few had ever had a long-term relationship [5], though marriage or cohabitation was slightly more common among the women. Altogether, the outcome must be considered rather poor, taking the high intellectual ability of the group into account.

## Conclusion

ASDs in adulthood may be diagnosed according to criteria reflecting the same triad of socio-communicative restrictions as in children. A wide range of symptoms will be found in all ASD subgroups, questioning the current classification. Patterns of comorbidity are insufficiently described in adult patients with ASD. This study demonstrated the high rates of DSM-IV axis I and II disorders, especially depression and ADHD. Differences between men and women were very few. Our results reflect the indistinct demarcations of the adult clinical neurodevelopmental phenotypes and stress the importance of the clinician's attention to a wide spectrum of psychiatric symptoms. These findings point to the need for a careful reexamination of the exclusion criteria of concomitant disorders for adult patients with ASDs in the next revision of the DSM. In spite of a normal or high intelligence, many subjects with adult ASD have considerable psychosocial impairment.

## Limitations

This study has a number of limitations. First, the lack of comparison group. All subjects were, however, consecutively recruited, which gives the study group a representative quality. Second, in order to obtain a reasonable study group size, two groups of patients from different sites were pooled. The groups from the two sites were investigated with almost, but not exactly, the same protocol. The two groups were, however, fairly similar in important variables such as age, sex, and intellectual level.

Both study sites have been involved in a common genetic project, and methods for assessment of subjects with ASDs were established in 1990s. Still, frequencies of disorders differed between sites: whereas subjects with AD were rare in both sites, the frequency of Gothenburg subjects with AS almost equaled that with PDD NOS, but the large majority of the Paris subjects were diagnosed with AS.

Our study group is most likely representative of clinical patients in adult psychiatry, though some prevalences of comorbid psychiatric symptoms may have been overestimated due to the fact that many of these patients had earlier psychiatric contacts. There is a need for population-based studies of ASDs and their overlapping conditions in adults.

## Competing interests

The authors declare that they have no competing interests.

## Authors' contributions

BH, PC, HA,OS, CG, MR and ML designed this study and its protocols. BH, RD, PC, AN, EW, OS, EH, AS, HA, MR and ML collected data through their clinical work. BH performed the statistical analyses and wrote the manuscript together with RD, HA, CG, MR and ML. All authors read and approved the final manuscript.

## Pre-publication history

The pre-publication history for this paper can be accessed here:


